# How I do it: endoscopic endonasal transclival resection of ventral foramen magnum meningioma

**DOI:** 10.1007/s00701-026-06941-0

**Published:** 2026-06-11

**Authors:** Yen-Yu Lin, Chien-Fu Yeh, Ming-Ying Lan, Wei-Hsin Wang

**Affiliations:** 1 Department of Neurosurgery, Neurological Institute, Taipei Veterans General Hospital, Taipei, Taiwan; 2 School of Medicine, National Yang Ming Chiao Tung University, Taipei, Taiwan; 3 Department of Otorhinolaryngology-Head and Neck Surgery, Taipei Veterans General Hospital, Taipei, Taiwan

**Keywords:** Endoscopic endonasal approach, Meningioma, Skull base, Foramen magnum

## Abstract

**Background:**

Surgical management of ventral foramen magnum meningiomas (FMMs) is challenging due to intervening neurovascular structures. The endoscopic endonasal transclival approach provides a direct access, offering a potential route to achieve Simpson Grade 1 resection while minimizing brainstem retraction.

**Method:**

We detail the technical nuances of a binostril, four-handed EEA for a ventral FMM. We explain the key anatomical landmarks required for safe decompression, including clival drilling limits, the identification of pre-medullary neurovascular structures, and the execution of a multilayered reconstruction.

**Conclusion:**

The EEA is a safe alternative for ventral FMMs in cases without significant lateral or caudal extension.

**Supplementary Information:**

The online version contains supplementary material available at 10.1007/s00701-026-06941-0.

## Introduction

**Fig. 1:**
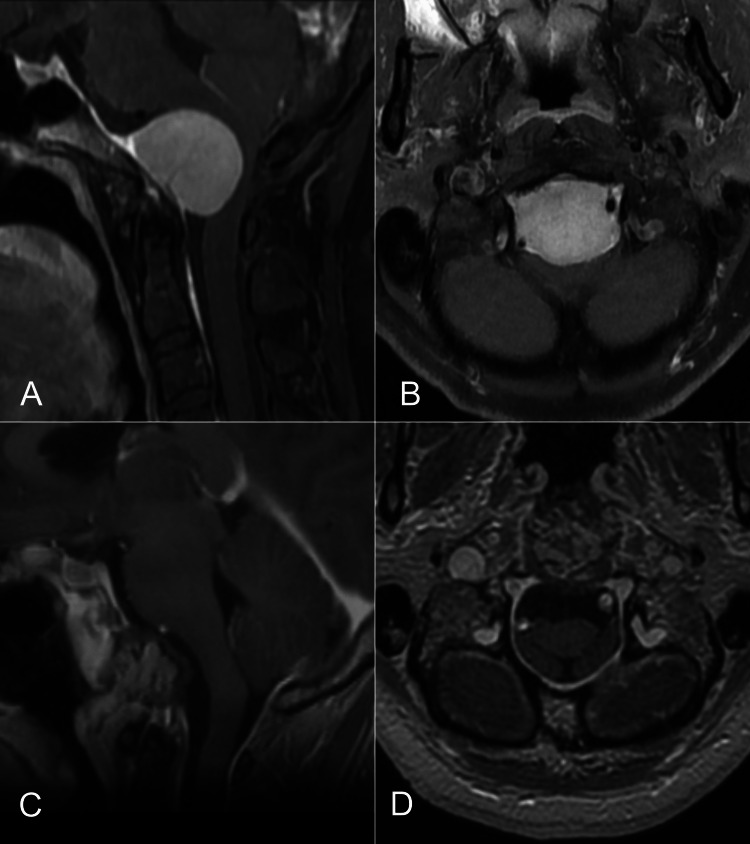
A representative case involves a 50-year-old female who presented with severe myelopathy and was diagnosed with a ventral foramen magnum meningioma. An endoscopic endonasal transclival approach was adopted, achieving Simpson grade I tumor resection. The patient experienced significant improvement in myelopathy symptoms postoperatively, with no new neurological deficits and no CSF leakage. She was able to return to her normal daily activities 2 weeks after the surgery. **A**, **B** Preoperative T1-weighted contrast-enhanced MRI: Sagittal (**A**) and axial (**B**) views demonstrate a large, ventral foramen magnum meningioma occupying the inferior clivus. The lesion causes significant posterior displacement of the brainstem and lateral displacement of the bilateral vertebral arteries. **C**, **D** Postoperative T1-weighted contrast-enhanced MRI: Sagittal (**C**) and axial (**D**) views 3 months after the surgery confirm total tumor removal and brainstem decompression. The reconstruction site shows a well-positioned vascularized flap and fat graft obliterating the bony defect

**Fig. 2:**
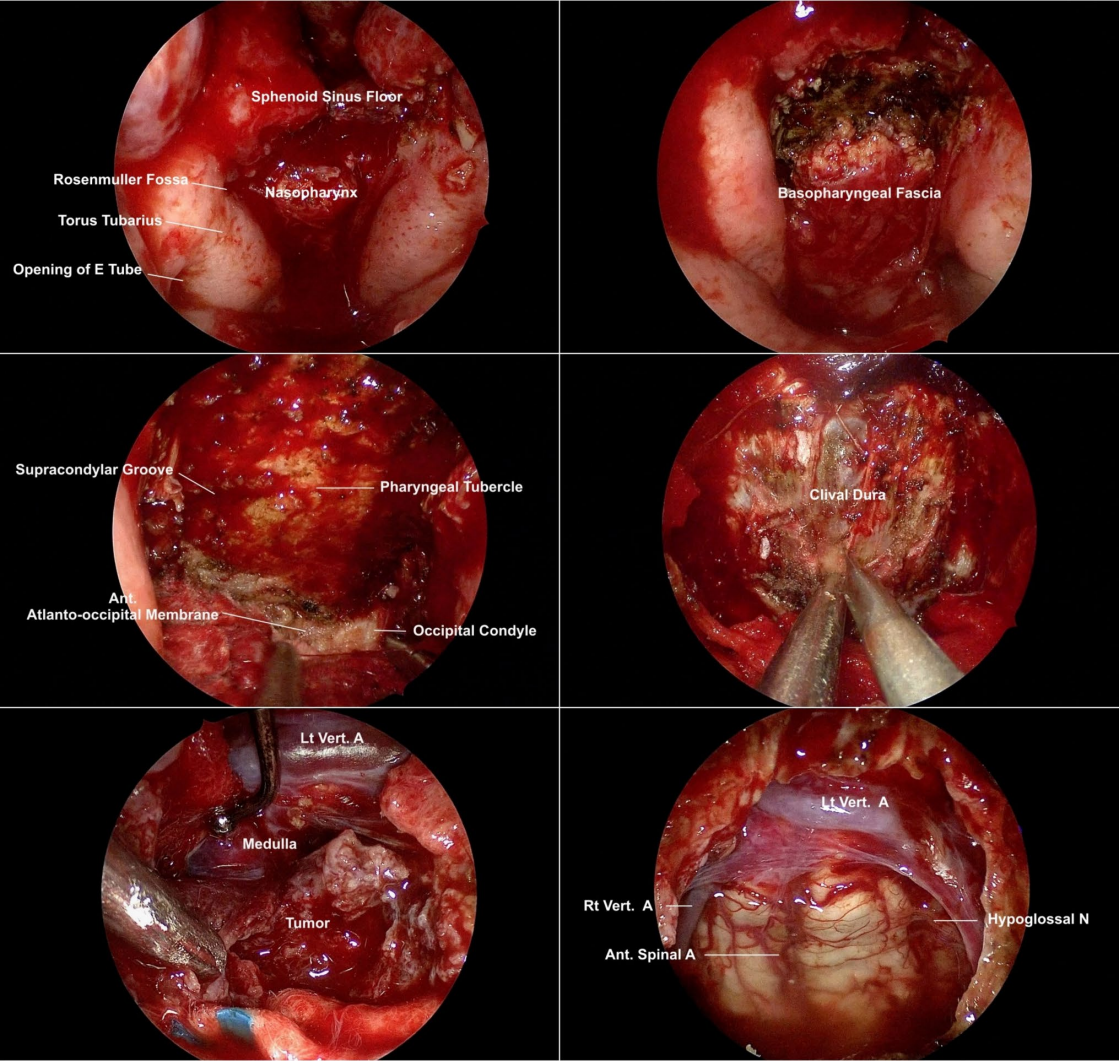
Intraoperative endoscopic steps and surgical anatomy: **A** Endoscopic view of the nasopharynx following sphenoidotomy and maxillary crest flattening, providing the initial surgical corridor. **B** Delineation of the inverted U-shaped nasopharyngeal flap, with bilateral borders extending along the Rosenmüller fossa. **C** Visualization of the inferior clivus, foramen magnum, and medial occipital condyles following the reflection of the nasopharyngeal flap. **D** Dural incision is made along the midline after adequate exposure of the dura. **E** Precise microsurgical dissection of the tumor from the neurovascular structures along the arachnoid plane. **F** Post-resection view confirming Simpson grade 1 tumor removal with preservation of the bilateral vertebral arteries, hypoglossal nerve and the anterior spinal artery.(E tube = Eustachian tube, Hypoglossal N = Hypoglossal nerve, Vert. A = Vertebral artery)

Foramen magnum meningiomas (FMMs) are rare, representing only 1.8% to 3.2% of all intracranial meningiomas [1]. Surgical management of FMMs with ventral dural insertions remains a formidable challenge, as the brainstem and lower cranial nerves lie between the surgeon and the tumor when using traditional posterior or posterolateral corridors [2]. The endoscopic endonasal approach (EEA) has emerged as a direct, "pre-medullary" route to the lower clivus and ventral foramen magnum [3]. By utilizing this corridor, surgeons can achieve tumor resection while minimizing brainstem manipulation. We present the technical nuances and surgical anatomy relevant to the resection of FMMs via the EEA (Figs. 1 and 2).

## Relevant surgical anatomy

The surgical corridor for the inferior third of the clivus extends from the floor of the sphenoid sinus, inclining posteroinferiorly toward the foramen magnum. The lateral boundaries are superficially defined by the torus tubarius and the fossa of Rosenmüller, which serve as critical landmarks for the parapharyngeal internal carotid artery (ICA). The nasopharyngeal posterior wall consists of the mucosa, basopharyngeal fascia, and the prevertebral musculature, specifically the longus capitis and rectus capitis anterior muscles [5].

Deep to the musculature, the anterior atlanto-occipital membrane attaches to the anterior margin of the foramen magnum and the arch of the atlas (C1), overlying the apical ligament and the odontoid process. Key osseous landmarks of the inferior clivus include the pharyngeal tubercle—a midline prominence—and the supracondylar groove. The latter identifies the location of the hypoglossal canal, defining the safe lateral boundary for bone resection. The cortical bone surrounding this canal dictates the permissible depth of any necessary condylectomy [7]. Following the lower clival resection, the dura is exposed, providing access to the premedullary cistern. Within this space, the lower cranial nerves (CN IX, X, XI, and XII) and critical vasculature, including the vertebral arteries (VA), posterior inferior cerebellar arteries (PICA), and the anterior spinal artery, must be meticulously preserved [6].

## Description of the technique

The patient was placed in a supine position with the head secured in a Mayfield skull clamp. An endoscopic, binostril, four-handed technique was employed under the guidance of neuronavigation. The inferior two-thirds of the middle turbinate were resected, and an extended vascularized nasoseptal flap was harvested and stored. Following a wide sphenoidotomy and posterior septectomy, the midline maxillary crest was flattened to expand the surgical window toward the nasopharynx.

An inverted U-shaped incision was performed on the posterior nasopharyngeal wall using a Colorado needle, extending from the sphenoid sinus floor superiorly to the lateral boundaries defined by the petroclival fissure. The mucosa along with prevertebral fascia and musculature were stripped to expose the inferior clivus, the anterior margin of the foramen magnum, and the bilateral occipital condyles. Key landmarks, including the pharyngeal tubercle, supracondylar groove, and the anterior arch of the atlas, were identified.

High-speed drilling of the ventral inferior clivus was performed under navigation to achieve full exposure of the dural insertion. Venous bleeding from basilar plexus or inferior petrous sinus was controlled with hemostatic matrix and bipolar cautery. Following dural coagulation, a vertical durotomy was performed along the midline. Intratumoral debulking was carried out using tumor forceps and an ultrasonic aspirator (CUSA). Microsurgical dissection of the tumor was initiated at bilateral margins until the arachnoid plane and the VAs were visualized. Tumor removal proceeded along this plane using sharp dissection to prevent traction injury to the lower cranial nerves and delicate perimedullary perforating vessels. A Simpson Grade I resection was achieved, and a 45-degree endoscope was utilized to inspect the lateral and inferior recesses for residual tumor.

Multi-layered skull base reconstruction was performed. An inlay of collagen matrix (DuraGen Plus) and fascia lata were placed to repair the dural defect; a fat graft was then used to obliterate the bony dead space. The vascularized nasoseptal flap was rotated to cover the surgical field, with edges secured using a dural sealant. A lumbar drain was placed to modulate cerebrospinal fluid (CSF) pressure.

## Indications

The EEA is particularly suited for ventrally located FMMs and lower cranial nerve schwannomas. The midline pre-medullary corridor provides direct access to the dural base, enabling early devascularization while avoiding brainstem retraction and neurovascular transposition.

## Limitations

1. Caudal Extension: Resections extending below the atlas arch may require a transoral approach, which can increase the challenge of reconstruction and the risk of CSF leakage.
2. Lateral Limits: Lesions extending significantly lateral to the hypoglossal canals are constrained by the hypoglossal nerves, often necessitating a far-lateral approach.
3. Vascular Encasement: For tumors encasing the VA, the lack of initial vascular control significantly increases the risk of irreversible hemorrhagic complications.

## How to avoid complications

1. Strategic Patient Selection**:** Detailed preoperative imaging is mandatory to evaluate the tumor's relationship to the VA and lower cranial nerves. If the lesion extends lateral to the hypoglossal canal, demonstrates encasement of the VA or shows loss of cortical cuff between the tumor and the brainstem, traditional transcranial corridors should be prioritized.
2. Neurophysiological Protection**:** Intraoperative neuromonitoring (IONM), specifically for cranial nerves IX through XII, provides real-time feedback during dissection, reducing the risk of iatrogenic injury due to traction.
3. Multilayered Reconstruction: The large volume of clival bone loss during exposure makes flap attachment challenging. A robust multilayered reconstruction is critical. A fat graft used to obliterate the bony dead space can help prevent brainstem herniation. A wider nasal septal flap, with careful downward rotation to cover the inferior border of the defect, is a key step [4].
4. Postoperative Management: Considering the intraoperative high-flow CSF leak, the use of a lumbar drain is recommended to facilitate flap adherence. Aggressive postoperative nasal hygiene and nasal inspection are also required to monitor the flap’s condition and prevent secondary rhinosinusitis.

## Specific information for the patient

Patients should be counseled on the risks and benefits of various surgical corridors. Disclosure must include the potential for catastrophic vascular injury and lower cranial nerve deficits. The risk of CSF rhinorrhea should be emphasized, as it may necessitate prolonged hospitalization, secondary repair, or increase the risk of meningitis.

## 10 key points

1. The EEA provides a direct route to the dural base of ventral FMMs allowing for early devascularization while avoiding brainstem retraction.
2. Ideal candidates are patients with ventral FMM who have a preserved cortical cuff and do not extend caudally beyond the C1 anterior arch or laterally beyond the hypoglossal canals.
3. A two-surgeon, four-handed technique is essential for dynamic visualization and meticulous bimanual dissection.
4. An extended nasoseptal flap must be harvested and protected to ensure high-quality reconstruction.
5. The petroclival fissure, pharyngeal tubercle, occipital condyles, and supracondylar grooves are useful landmarks during clival bone drilling.
6. Precise localization of the VA and their relationship to the tumor is essential to prevent vascular injury.
7. Utilizing ultrasonic aspiration for internal debulking, followed by sharp dissection to release the tumor from the arachnoid plane, is performed similarly to microsurgical techniques.
8. Reconstruction includes an inlay collagen matrix, an onlay fascia lata, a fat graft to obliterate dead space. The nasopharyngeal flap is then repositioned to cover the inferior border.
9. Postoperative lumbar drainage for 5–7 days is recommended to reduce pressure on the repair site and facilitate flap adherence.
10. Follow-up with contrast-enhanced MRI is necessary to monitor tumor control and ensure flap viability.

## Supplementary Information

Below is the link to the electronic supplementary material.

ESM 1 Supplementary Material 1 (MP4 549 MB)

## Data Availability

No datasets were generated or analysed during the current study.

## Abbreviations

CSF: Cerebrospinal fluid
EEA: Endoscopic endonasal approach
FMM: Foramen magnum meningioma
ICA: Internal carotid artery
IONM: Intraoperative neuromonitoring
MRI: Magnetic resonance imaging
PICA: Posterior inferior cerebellar artery
VA: Vertebral artery
